# Acute Carpopedal Spasm During Medical Termination of Pregnancy: A Case of Hyperventilation-Induced Hypocalcemia

**DOI:** 10.7759/cureus.99667

**Published:** 2025-12-19

**Authors:** Taif Makhafah, Farah Almoussa, Abdulmalik Alaqeeli, Inas Babic, Hassan Allam

**Affiliations:** 1 College of Medicine, Alfaisal University, Riyadh, SAU; 2 Obstetrics and Gynecology, Prince Sultan Military Medical City, Riyadh, SAU; 3 Obstetrics and Gynecology, King Abdulaziz University Faculty of Medicine, Rabigh, SAU

**Keywords:** carpopedal spasm, hyperventilation, hypocalcemia, medical termination of pregnancy, misoprostol, missed abortion, neuromuscular symptoms, pregnancy loss, respiratory alkalosis

## Abstract

Acute neuromuscular symptoms during medical management of pregnancy loss are rare and may mimic life-threatening obstetric or neurologic emergencies. A 40-year-old multigravida woman at 14 weeks and 5 days of gestation was admitted for medical management of a missed abortion using sublingual misoprostol. Three hours after administration, she developed acute bilateral carpopedal spasms accompanied by hyperventilation, chest discomfort, agitation, and perioral numbness. Laboratory evaluation revealed marked respiratory alkalosis with reduced ionized calcium, while endocrine workup, including parathyroid hormone, vitamin D, and magnesium levels, was normal. Extensive assessment excluded uterine rupture, pulmonary embolism, sepsis, and seizure activity. Symptoms improved after intravenous calcium administration and resolved completely following expulsion of the gestational sac. This case highlights hyperventilation-induced respiratory alkalosis as a reversible cause of acute neuromuscular symptoms during medical management of pregnancy loss, underscoring the importance of prompt recognition, targeted treatment, and avoidance of unnecessary interventions.

## Introduction

Medical management of second-trimester pregnancy loss commonly involves the use of misoprostol, a prostaglandin E1 analog that is widely recommended by international guidelines because of its efficacy, availability, and favorable safety profile. Although misoprostol is generally well tolerated, its use is frequently associated with uterine cramping, gastrointestinal symptoms, and autonomic responses such as anxiety and tachycardia [1,2]. Acute physiologic stress and pain during pregnancy termination may precipitate hyperventilation, which, in turn, can lead to clinically significant respiratory alkalosis. In this setting, alkalosis increases calcium binding to albumin, resulting in a rapid decrease in ionized calcium levels without a true reduction in total body calcium [2-4].

Symptomatic hypocalcemia due to respiratory alkalosis is an uncommon but well-recognized cause of acute neuromuscular irritability, manifesting as perioral numbness, carpopedal spasm, or tetany, and may mimic obstetric, cardiopulmonary, or neurologic emergencies [1,3]. In pregnant patients undergoing medical termination, such presentations may prompt concern for life-threatening conditions, including pulmonary embolism, uterine rupture, or seizure disorders. Awareness of this reversible biochemical mechanism is essential for timely diagnosis, appropriate treatment, and avoidance of unnecessary interventions.

## Case presentation

A 40-year-old multigravida woman (gravida 9, para 7+1) at 14 weeks and 5 days of gestation was admitted for medical management of a nonviable pregnancy. First-trimester ultrasonography demonstrated a blighted ovum consistent with a missed abortion. At admission, the patient reported no abdominal pain, vaginal bleeding, dyspnea, chest pain, fever, or neurologic symptoms.

Her medical history was unremarkable, with no known metabolic or neurologic disorders and no medication use. She had no known drug allergies. Surgical history included one prior cesarean delivery and one dilation and curettage performed for abortion-related bleeding. Her obstetric history included one spontaneous vaginal delivery, one cesarean delivery followed by five successful vaginal births after cesarean, and one prior first-trimester miscarriage.

On admission, vital signs were within normal limits. Physical examination was unremarkable. Baseline laboratory studies, including complete blood count and routine serum biochemistry, were within reference ranges. Medical termination was initiated with a first dose of 600 μg of sublingual misoprostol.

Approximately three hours after misoprostol administration, the patient acutely developed bilateral carpopedal spasms characterized by sustained tonic contraction of the hands and feet. These were accompanied by marked hyperventilation, palpitations, chest discomfort, agitation, and perioral numbness. She remained conscious and oriented to person, place, and time.

At symptom onset, vital signs revealed a heart rate of 120 beats per minute, blood pressure of 142/67 mmHg, respiratory rate of 20 breaths per minute, oxygen saturation of 82% on room air, and a temperature of 37.1 °C. A medical emergency response (Code Green) was activated. Supplemental oxygen was administered via face mask at 5 L/minute.

Urgent laboratory testing, including complete blood count, coagulation profile, serum electrolytes, and venous blood gas analysis, was obtained. Venous blood gas analysis demonstrated marked respiratory alkalosis with a pH of 7.57, partial pressure of carbon dioxide of 21 mmHg, bicarbonate level of 19.2 mmol/L, and an elevated lactate level of 7.6 mmol/L. Ionized calcium was reduced to 1.2 mmol/L. Chest auscultation revealed equal bilateral air entry without wheezing or crackles. Abdominal examination was benign, and pelvic examination revealed no vaginal bleeding.

The rapid response team evaluated the patient. The initial differential diagnosis included acute symptomatic hypocalcemia, pulmonary embolism, uterine rupture, sepsis, and seizure activity. An electrocardiogram showed sinus tachycardia with a normal QTc interval. A portable chest radiograph showed no acute cardiopulmonary abnormalities. Bedside ultrasonography demonstrated an intact uterus without evidence of rupture and no free fluid in the pouch of Douglas.

The patient received 1 L of intravenous normal saline and 10 mmol of intravenous calcium gluconate. Misoprostol administration was withheld, and she was transferred to the high-dependency unit for continuous monitoring and strict input-output charting.

Approximately three hours later, the patient experienced a second episode of bilateral carpopedal spasm, coinciding with the onset of vaginal bleeding and complete expulsion of the gestational sac. The neuromuscular symptoms resolved completely following expulsion of the products of conception. Speculum examination revealed no active bleeding. The expelled tissue was sent for histopathologic examination. Repeat bedside ultrasonography demonstrated an empty uterine cavity with an endometrial thickness of 1.4 cm.

Repeat venous blood gas analysis showed a persistent reduction in ionized calcium (1.07 mmol/L; corrected calcium, 1.1 mmol/L), and a second dose of intravenous calcium gluconate was administered. An endocrinology consultation was obtained. Further evaluation, including serum parathyroid hormone, vitamin D, and magnesium levels, yielded results within normal reference ranges.

The patient remained hemodynamically stable with no recurrence of symptoms. She was discharged the following day. Given the transient nature of the bilateral spasms and preserved consciousness, an epileptogenic cause was considered unlikely; however, outpatient neurology follow-up was arranged to definitively exclude a seizure disorder.

## Discussion

This case describes an acute episode of bilateral carpopedal spasm occurring during medical management of a second-trimester missed abortion, ultimately attributable to respiratory alkalosis-induced reduction in ionized calcium rather than a primary calcium disorder or obstetric complication. The temporal relationship between misoprostol administration, onset of hyperventilation and neuromuscular symptoms, biochemical evidence of marked alkalosis, and resolution following correction of calcium levels and completion of abortion supports this interpretation [1-5]. Importantly, normal parathyroid hormone, vitamin D, and magnesium levels argue against underlying disorders of calcium metabolism and indicate a functional, transient disturbance.

Hyperventilation leads to hypocapnia and an increase in blood pH, which enhances the binding of calcium to albumin and reduces the fraction of biologically active ionized calcium. Even modest reductions in ionized calcium can increase neuromuscular excitability, resulting in perioral paresthesia, carpopedal spasm, and tetany, despite normal total serum calcium concentrations. In this patient, the profound respiratory alkalosis (pH 7.57) likely precipitated a rapid decline in ionized calcium sufficient to cause clinically significant symptoms. The associated elevation in lactate may reflect increased muscular activity and adrenergic stimulation during the acute episode [6-10].

The clinical presentation raised concern for several life-threatening conditions, including pulmonary embolism, uterine rupture, sepsis, and seizure activity. Pulmonary embolism was considered unlikely given the absence of chest imaging abnormalities, rapid symptom resolution, and lack of persistent hypoxemia. Uterine rupture was excluded by bedside ultrasonography and clinical stability. Sepsis was improbable in the absence of fever, leukocytosis, or hemodynamic instability. An epileptogenic process was also unlikely given preserved consciousness, symmetric involvement, absence of postictal confusion, and biochemical abnormalities consistent with a metabolic cause. Recognition of respiratory alkalosis-induced hypocalcemia was therefore critical in narrowing the differential diagnosis and guiding appropriate management.

Although misoprostol has not been directly associated with hypocalcemia or neuromuscular excitability, its known effects, including uterine contractions, pain, anxiety, and autonomic activation, may indirectly precipitate hyperventilation in susceptible patients. This case underscores the importance of close monitoring during medical termination, particularly in second-trimester pregnancies, where physiologic stress may be more pronounced. Prompt assessment of acid-base status and ionized calcium levels should be considered when acute neuromuscular symptoms develop in this setting.

## Conclusions

This case emphasizes that acute hyperventilation during medical management of pregnancy loss can precipitate respiratory alkalosis with a rapid reduction in ionized calcium, resulting in transient neuromuscular symptoms that may mimic serious obstetric or neurologic emergencies. Early recognition of this physiologic mechanism, supported by timely blood gas and electrolyte assessment, is essential to guide appropriate management, avoid unnecessary interventions, and ensure patient safety during medical termination of pregnancy.
